# Neuroanatomical changes associated with age-related hearing loss and listening effort

**DOI:** 10.1007/s00429-020-02148-w

**Published:** 2020-09-22

**Authors:** Stephanie Rosemann, Christiane M. Thiel

**Affiliations:** 1 Biological Psychology, Department of Psychology, Department for Medicine and Health Sciences, Carl-von-Ossietzky Universität Oldenburg, Ammerländer Heerstraße 114-118, 26111 Oldenburg, Germany; 2 Cluster of Excellence “Hearing4all”, Carl von Ossietzky Universität Oldenburg, Oldenburg, Germany

**Keywords:** Ageing, Hearing loss, Listening effort, Grey matter volume, Cortical thickness, Diffusion tensor imaging

## Abstract

Age-related hearing loss is associated with a decrease in hearing abilities for high frequencies and therefore leads to impairments in understanding speech—in particular, under adverse listening conditions. Growing evidence suggests that age-related hearing loss is related to various neural changes, for instance, affecting auditory and frontal brain regions. How the decreased auditory input and the increased listening effort in daily life are associated with structural changes is less clear, since previous evidence is scarce and mostly involved low sample sizes. Hence, the aim of the current study was to investigate the impact of age-related untreated hearing loss and subjectively rated daily life listening effort on grey matter and white matter changes in a large sample of participants (*n* = 71). For that aim, we conducted anatomical MRI and diffusion tensor imaging (DTI) in elderly hard-of-hearing and age-matched normal-hearing participants. Our results showed significantly lower grey matter volume in the middle frontal cortex in hard-of-hearing compared to normal-hearing participants. Further, higher listening effort was associated with lower grey matter volume and cortical thickness in the orbitofrontal cortex and lower grey matter volume in the inferior frontal cortex. No significant relations between hearing abilities or listening effort were obtained for white matter integrity in tracts connecting auditory and prefrontal as well as visual areas. These findings provide evidence that hearing impairment as well as daily life listening effort seems to be associated with grey matter loss in prefrontal brain regions. We further conclude that alterations in cortical thickness seem to be linked to the increased listening effort rather than the hearing loss itself.

## Introduction

Age-related hearing loss—also termed presbycusis—is one of the most prevalent chronic disorders affecting older adults. It involves the decrease in hearing abilities for high frequencies and therefore leads to impairments in understanding and processing speech, particularly in difficult listening situations (Cardin 2016; Lin 2012). Moreover, age-related hearing loss is associated with an increased listening effort (Matthen 2016; Pichora-Fuller et al. 2016; Rosemann and Thiel 2018; Rudner 2016). Listening is defined as the process of hearing with intention and attention (Kiessling et al. 2003), whereas the term listening effort refers to the mental effort, such as the allocation of attentional as well as cognitive resources, in demanding (listening) environments (Bernarding et al. 2017; Pichora-Fuller et al. 2016). An increase in listening effort may decrease resources available for other cognitive operations (Humes et al. 2013a). Hence, effortful listening plays not only a role in age-related hearing loss, but also in normal-hearing individuals during acoustically adverse listening situations (Erb and Obleser 2013; Pichora-Fuller et al. 2016; Wild et al. 2012; Wong et al. 2009). Growing evidence suggests that age-related hearing loss is associated with decreased cognitive functioning (Armstrong et al. 2018; Peele et al. 2011; Rosemann and Thiel 2020) along with neural alterations, for instance, changes in task-related brain activity in auditory (Berding et al. 2015; Campbell and Sharma 2013, 2014; Peele et al. 2011; Wong et al. 2009) and frontal regions (Berding et al. 2015; Campbell and Sharma 2013, 2014; Erb and Obleser 2013; Peele et al. 2011; Rosemann and Thiel 2018; Wong et al. 2009). Further, decreases in resting state functional connectivity in frontal areas were linked to increased listening effort in elderly participants (Rosemann and Thiel 2019) and sensorineural hearing loss (Luan et al. 2019a).

However, how grey and white matter changes accompany age-related hearing loss and increasing listening effort is less understood. Previous studies revealed an association between high-frequency hearing loss and lower grey matter volume in the auditory cortex (Armstrong et al. 2018; Eckert et al. 2012, 2019; Qian et al. 2017) and accelerated decline in whole-brain volume (Eckert et al. 2019; Lin et al. 2014; Peele et al. 2011; Qian et al. 2017; Rigters et al. 2017). Furthermore, grey matter loss was found in the superior and medial frontal gyri in participants with hearing loss compared to normal-hearing individuals (Husain et al. 2011). Boyen et al. (2013) showed grey matter increases in the temporal cortex (BA 22), but also decreases in grey matter in the frontal cortex, occipital lobe and hypothalamus in hard-of-hearing compared to normal-hearing participants. However, both studies aimed to differentiate grey matter volume differences between tinnitus and hearing loss and sample size was rather low (*n* = 7 and *n* = 16, respectively). Complementing previous work on hearing abilities, cortical thickness in the prefrontal cortex was positively related to speech perception in older adults (Giroud et al. 2018; Wong et al. 2010). Additionally, grey matter decreases in the superior temporal, frontal, insular and hippocampal brain regions were associated with poorer speech in noise understanding in older participants (Rudner et al. 2019).

With respect to diffusion tensor imaging (DTI), the role of hearing loss is even less clear since only a few studies have investigated white matter changes in hard-of-hearing participants. Two studies investigated changes in white matter integrity and found significantly lower fractional anisotropy (FA) in hard-of-hearing compared to normal-hearing participants in longitudinal and fronto-occipital tracts (Husain et al. 2011; Luan et al. 2019b). However, in the former study, the sample size was rather low and the statistical threshold liberal, whereas the latter study also covered congenital, noise-induced and drug-induced hearing impairments and was not restricted to an age-related decline in hearing abilities. Another DTI study identified increased diffusivity, but decreased FA in auditory areas as well as increased diffusivity in language-related areas in participants with age-related hearing loss (Ma et al. 2016). Sample size was relatively low in this study as well (*n* = 15). In contrast, Profant et al. (2014) found no associations between hearing impairment and grey or white matter changes. However, this study did not include a normal-hearing control group; presbycusis participants with mild and severe hearing impairments were compared to each other.

To conclude, the evidence regarding neuroanatomical differences between hard-of-hearing and age-matched normal-hearing individuals is scarce and most of the previous studies encompassed relatively low sample sizes. Further, previous work did not focus exclusively on age-related hearing loss and the influence of increased listening effort, as is often experienced by hard-of-hearing participants, on structural brain alterations has not been investigated so far. In contrast to pure-tone thresholds, a measure of listening effort involves the assessment of cognitive resource allocation during speech processing. Thus, listening effort may be a more sensitive marker of neural alterations than hearing abilities determined by pure-tone audiometry (Bernarding et al. 2017; Pichora-Fuller et al. 2016). Therefore, the aim of the current study was to investigate the impact of untreated age-related hearing loss and self-rated daily life listening effort on grey and white matter changes in a large sample of participants (*n* = 71) covering normal-hearing participants as well as individuals with mild to moderate age-related hearing loss. For that aim, anatomical MRI and diffusion tensor imaging (DTI) in elderly hard-of-hearing and age-matched normal-hearing participants were analysed to assess grey matter volume, cortical thickness and white matter integrity.

Most previous studies assessed grey matter volume with respect to hearing loss; however, there is evidence that cortical thinning in the auditory cortex is closely linked to presbycusis as well as speech perception and may thus be a more adequate indicator of structural changes in age-related hearing loss (Giroud et al. 2018). In addition, cortical thickness seems to be a valuable measure for detecting subtle changes in brain atrophy that may arise prior to degeneration in the frontal and temporal lobe, such as in early differentiation between frontotemporal dementia and mild cognitive impairment (Hartikainen et al. 2012). The authors of this study argue that cortical thickness assessment might be used as a marker of atrophy even at the single-subject level when comparing between frontotemporal dementia and mild cognitive impairment. Moreover, cortical thickness is also discussed as a biomarker in Alzheimer’s disease (Bakkour et al. 2009; Dickerson and Wolk 2012). The assessment of cortical thickness may therefore be of interest as additional neural measure to support the development of treatment or rehabilitation options in age-related hearing loss. White matter integrity—as supposed to reflect axon damage or demyelination—has previously gained only minute attention with respect to research in hearing loss (Ma et al. 2016), whereas its role in age-related decline and cognitive functioning in healthy adults has been already acknowledged (Madden et al. 2009, 2013). Further, the plastic nature of white matter integrity, for instance, in response to cognitive training shown by Lövdén et al. (2010), may offer valuable implications for diagnosing and treating age-related cognitive disorders, which might also apply to age-related hearing loss (Madden et al. 2013).

Accordingly, the current study evaluated grey matter volume, cortical thickness as well as white matter integrity in order to disentangle structural changes in age-related hearing loss. Previous research consistently showed lower grey matter volume and cortical thickness in prefrontal and auditory brain regions as well as lower white matter integrity, indexed by FA values in fronto-occipital and longitudinal tracts associated with hearing loss and speech perception. Hence, our hypotheses were that (i) grey matter volume and cortical thickness are lower in the temporal and prefrontal brain regions in hard-of-hearing compared to normal-hearing participants (Boyen et al. 2013; Giroud et al. 2018; Husain et al. 2011; Rudner et al. 2019; Wong et al. 2010). Further, (ii) grey matter volume and cortical thickness in the prefrontal cortex should be negatively associated with self-rated daily life listening effort such that with high experienced listening effort there is a thinning of cortical thickness and loss of grey matter volume (Giroud et al. 2018; Rudner et al. 2019; Wong et al. 2010). Regarding white matter integrity, we expected (iii) decreased FA values in association tracts connecting auditory and prefrontal as well as visual areas—for instance, superior and inferior fronto-occipital and longitudinal fasciculi as well as in cingulum and uncinate fasciculus—in hard-of-hearing compared to normal-hearing participants (Husain et al. 2011; Luan et al. 2019b; Ma et al. 2016). Similarly, we hypothesized (iv) a negative association between white matter integrity in the aforementioned regions and self-rated listening effort, i.e. lower FA values with increased listening effort (Rosemann and Thiel 2019).

## Methods

### Participants

**Fig. 1:**
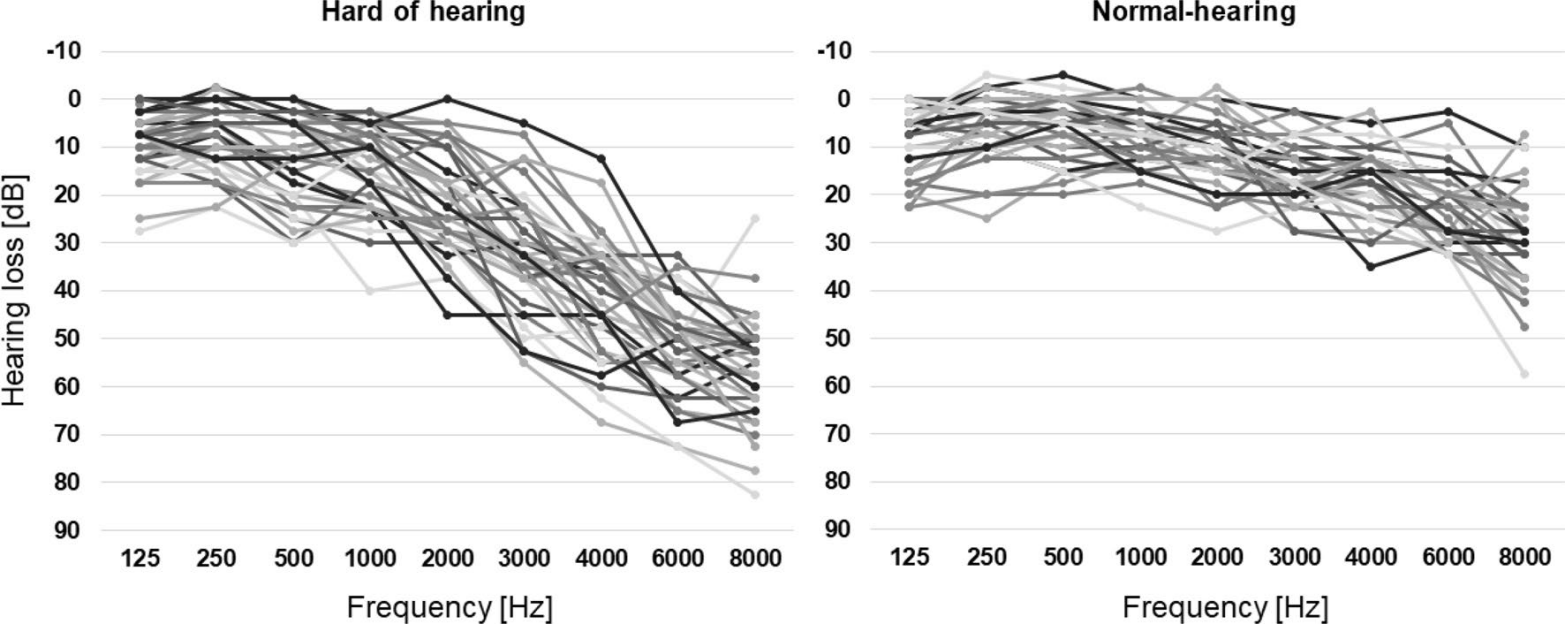
Individual pure-tone audiograms for hard-of-hearing (left) and normal-hearing (right) participants averaged over both ears

We analysed the structural MRI and DTI data from 38 hard-of-hearing and 33 normal-hearing participants that participated in functional MRI studies within the framework of the Cluster of Excellence Hearing4all. Functional data from these studies have already been published (Rosemann and Thiel 2018,2019,2020; Rosemann et al. 2020). The age range for recruitment was restricted to 50–75 years, and hard-of-hearing and normal-hearing subjects were matched in terms of age and gender. Data sets of participants that partook in more than one study were only included once (the earlier measurement). Two participants (in the hard-of-hearing group) did not have DTI data, but were included in the voxel-based morphometry (VBM) analysis. The mean age was 64.1 (± 5.6) years for hard-of-hearing participants and 62.9 (± 5.0) years for normal-hearing participants. The age range was 51–74 years. Age did not significantly differ between the groups (*p* > 0.1). Subjects with low-frequency hearing loss were excluded, i.e. hearing abilities for all participants were 30 dB HL or better for frequencies up to 1 kHz (except one value for one participant that was 40 dB at 1 kHz in one ear). High-frequency hearing loss was defined by more than 30 dB HL for at least three frequencies between 2 and 8 kHz. The hard-of-hearing individuals showed a varying bilateral sloping high-frequency hearing loss that was mild to moderate, symmetrical (≤ 15 dB difference between left and right ear for more than three frequencies) and only affecting high frequencies (≥ 2 kHz). Normal-hearing abilities were defined as 30 dB HL or better for each octave frequency between 125 and 3000 Hz and less than 30 dB HL for the mean value over 2000, 4000, 6000 and 8000 Hz (cf. WHO, 2001 definition of hearing loss; von Gablenz and Holube, 2015). Hearing thresholds for high frequencies (mean 2–8 kHz) were 40.28 (± 8.19) dB in the hard-of-hearing and 19.27 (± 6.03) dB in the normal-hearing group. Individual pure tone audiograms averaged over both ears for the two groups are depicted in Fig. 1. None of the participants reported current or previous use of a hearing aid. All participants were right-handed. Exclusion criteria for participation were previous or current psychiatric or neurological disorders as well as tinnitus.

### Experimental procedure

Experimental sessions started with a pure tone audiometry of the frequencies (125, 250, 500, 1000, 2000, 3000, 4000, 6000 and 8000 Hz) that was conducted in a soundproof chamber. MRI measurements were conducted in two sessions separated by a short break outside the scanner. In the first run, the functional MRI was measured. After the break, a resting state MRI, anatomical MRI and a diffusion MRI measurement were conducted. Functional MRI results and resting state analyses have been previously published (Rosemann and Thiel 2018,2019,2020). Outside the MRI and after a short break, participants filled out the listening effort questionnaire (“Höranstrengungsbogen”; Schulte et al. 2015). Participants could fill out the questionnaire themselves at their own pace. Further, participants were encouraged to ask questions whenever they had one. The listening effort questionnaire describes 17 listening situations of different difficulty in everyday life (e.g. ‘You meet some friends in a café and are able to see them during the conversation’ referring to medium listening situation or ‘You are watching the news on TV in a quiet environment’ referring to an easy listening situation). For every scenario, participants are asked to rate the listening effort they need in the different situations, for instance ‘How effortful is it to follow the conversation?’ All rating scales were 11-point Likert scales between 0 (not effortful at all) and 10 (extremely effortful). The mean rating value across all 17 situations was used as a marker of listening effort. Data for one participant was missing in this respective questionnaire. An additional analysis evaluated the listening effort separately for questions describing easy (no noise, four questions), medium (background noise for instance in a café, nine questions) and difficult listening situations (disturbed speech like poor audio quality during a telephone call, four questions).

### Data acquisition

A 3T whole-body Siemens Magnetom Prisma MRI machine with a 20-channel head coil was used for all measurements. Data were acquired with slightly different MR sequences due to software updates. The sample measured with the first parameter set included 39 participants (20 hard-of-hearing/19 normal-hearing) and 32 participants were measured with parameter set two (18 hard of hearing/16 normal hearing). Structural images were acquired with 3-D T1-weighted MPRAGE sequences with (1) TR = 2300, TE = 4.16, slice thickness 1 mm, 176 sagittal slices or (2) TR = 2000, TE = 2.07, slice thickness 0.75 mm, 224 sagittal slices. Diffusion tensor measurements were acquired with a multi-directional diffusion weighting (MDDW) sequence with a *b*-factor of 1000 s/mm^2^ including 70 transversal slices of the whole brain (slice thickness 2 mm, voxel size = 2.0 × 2.0 × 2.0 mm^3^). Further parameters of the diffusion-weighted sequence were (1) TR = 6000 ms, TE = 52 ms, 64 directions, 11 non-diffusion images or (2) TR = 9400 ms, TE = 68 ms, 15 directions, 10 non-diffusion images.

### Data analysis

#### Grey matter analysis

Grey matter metrics were analysed using SPM12 (Welcome Department of Imaging Neuroscience, London, UK) and the CAT12 (Gaser and Dahnke 2016) toolbox for SPM. We performed a voxel-based morphometry (VBM) and cortical thickness analysis using the anatomical T1-weighted image. Preprocessing steps in CAT12 included normalization to the Montreal Neurological Institute (MNI) stereotactic space, segmentation into grey and white matter as well as cerebrospinal fluid and smoothing (Gaussian smoothing, full width half maximum = 8 mm for the VBM and full width half maximum = 15 mm for the cortical thickness analysis). During segmentation, grey matter volume and cortical thickness measures were computed. A projection-based thickness approach was used to estimate cortical thickness and to create the central cortical surface for both hemispheres separately. A quality check was applied between segmentation and smoothing. One participant (from the hard-of-hearing group) was excluded due to inhomogeneous data. The remaining 70 participants were used in the statistical analysis. In the VBM analysis, total intracranial volume was used for global scaling. On the group level, we performed between-group comparisons (hard-of-hearing versus normal-hearing participants) and a linear regression analysis across all participants investigating the effect of self-rated listening effort (using CAT12 for model specification and SPM12 for model estimation and statistical analysis; not corrected for hearing abilities). For all analyses, effects were determined to be significant when passing a threshold of *p* < 0.05 (FWE cluster size inference with *p* < 0.001 cluster-forming threshold). Analyses were conducted for the whole brain and the multiple comparison problem is dealt with by using family-wise error correction based on random field theory. Peak coordinates are reported in MNI space.

#### Diffusion data analysis

DTI data were analysed with ExploreDTI (Leemans et al. 2009). Preprocessing included signal drift and Gibbs ringing correction as well as subject motion correction, eddy currents and EPI distortions using the subjects’ anatomical image and the REKINDLE estimation method. The diffusion tensor was estimated by the REKINDLE robust method excluding outliers. Mean FA values were computed using the ICBM**-**Mori (Mori et al. 2005) and Automated Anatomical Labelling (AAL; Tzourio-Mazoyer et al. 2002) atlases. FA values from superior and inferior fronto-occipital and longitudinal fasciculi as well as in cingulum and uncinate fasciculus were computed for further analysis. Then, FA values were compared between normal-hearing and hard-of-hearing participants by two-sample *t* tests. Bonferroni correction was applied to correct for the number of tracts. Pearson's correlation served to assess the relationship between FA values and self-rated listening effort across all participants (two-tailed; Bonferroni-corrected; not corrected for hearing abilities). Data analysis was performed in SPSS Statistics 25 (IBM, Armonk, NY, USA).

## Results

### Behavioural data

The mean self-rated listening effort experienced in daily life was rated as rather mild with 3.0 (± 1.4) in hard-of-hearing participants and 2.4 (± 1.2) in normal-hearing participants. The maximum self-rated listening effort in the normal-hearing group was 4.82 and in the hard-of-hearing group 6.81. An additional analysis evaluated the listening effort separately for the three different categories easy, medium and difficult situations. The mean scores were 1.47 (± 1.1) for the easy, 3.29 (± 1.7) for the medium and 4.07 (± 1.9) for the difficult situations in the hard-of-hearing group. In normal-hearing listeners the mean scores were 0.97 (± 0.97) for easy, 2.64 (± 1.3) for medium and 3.38 (1.8) for the difficult situations. The repeated measures ANOVA for the mean values of the listening effort questionnaire as well as the different sub-categories revealed a significant difference between both groups (F(1,67) = 4.045, *p* = 0.048, ɲ2 = 0.057), and no interaction between listening effort sub-category and group (*p* > 0.1).

Hearing thresholds for high frequencies (mean 2, 3, 4, 6 and 8 kHz over both ears) were 40.28 (± 8.19) dB in the hard-of-hearing and 19.27 (± 6.03) dB in the normal-hearing group. The mean high-frequency hearing loss values significantly differed between groups (T(69) = 12.15; *p* < 0.001). Listening effort and hearing loss significantly correlated with each other across all participants (*r* = 0.297, *p* = 0.012).

### Grey matter volume and cortical thickness

To investigate the association of high-frequency hearing loss and listening effort with grey matter volume and cortical thickness, we performed between-group comparisons (hard of hearing versus normal hearing) and regression analyses across all participants (using the mean values from the listening effort questionnaire; not corrected for hearing loss). Analyses were conducted on the whole-brain level and effects were determined to be significant when passing a threshold of *p* < 0.05 (FWE cluster size inference with *p* < 0.001 cluster-forming threshold).

**Fig. 2:**
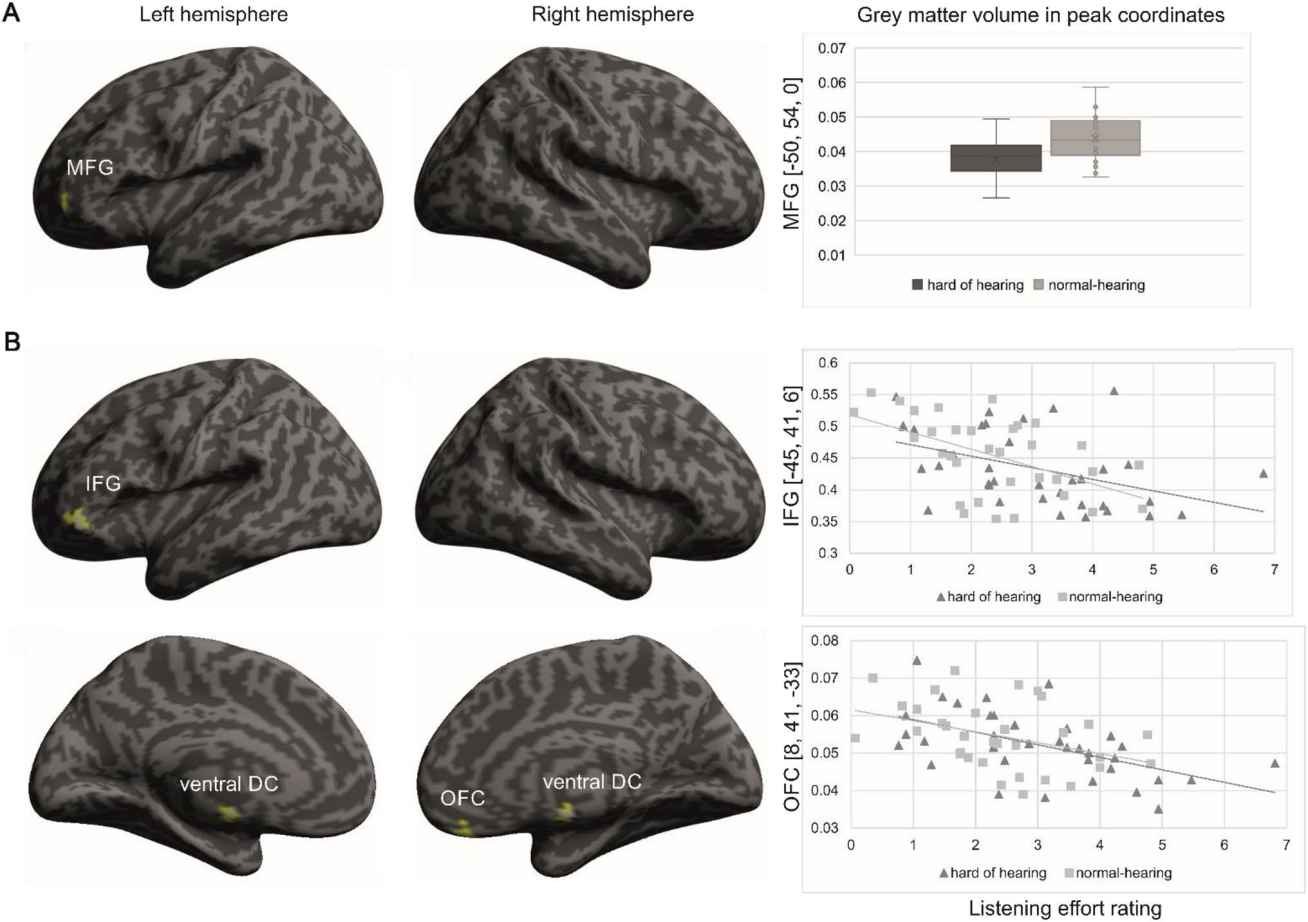
Grey matter volume associations with hearing loss and listening effort. **a** Significantly higher grey matter volume in normal-hearing compared to hard-of-hearing participants. **b** Significant correlation between grey matter volume and listening effort; *MFG* middle frontal gyrus, *IFG* inferior frontal gyrus, *OFG* orbitofrontal cortex [*p* < 0.05; FWE corrected on the cluster level]

Between-group comparisons showed significantly higher grey matter volume in the middle frontal gyrus (BA 46; MNI coordinate: *x* = − 50, *y* = 54, *z* = 0; cluster size *k* = 344; *z*-value = 3.95) in normal-hearing compared to hard-of-hearing participants (Fig. 2a). Cortical thickness did not significantly differ between both groups.

**Fig. 3:**
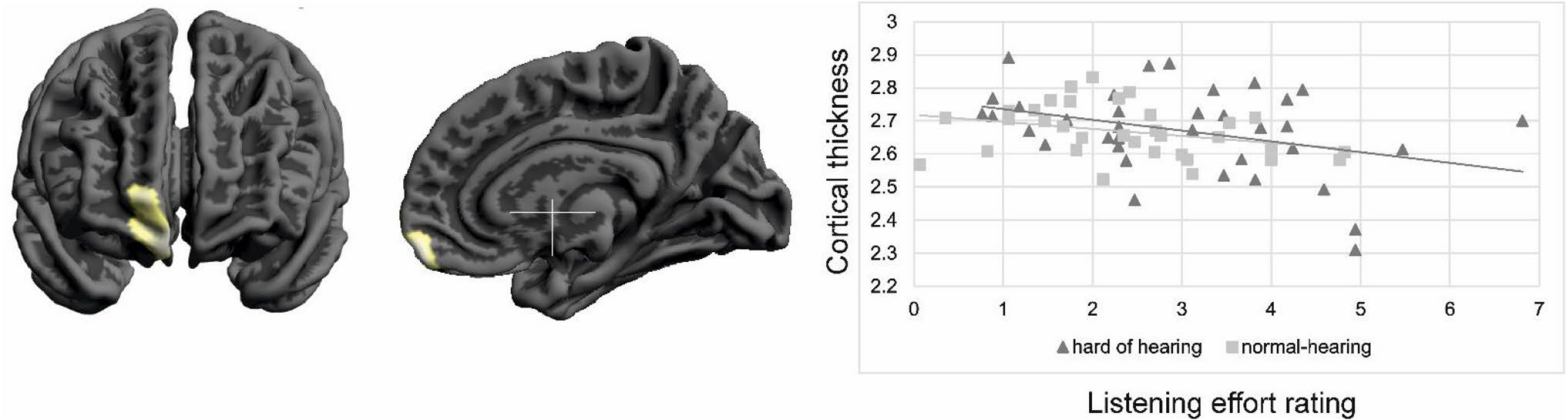
Significant correlation between cortical thickness in the right orbitofrontal cortex (BA 12) and listening effort [*p* < 0.05; FWE corrected on the cluster level]

Further, self-rated listening effort experienced in daily life was negatively associated with grey matter volume (Fig. 2b, Table 1) and cortical thickness (Fig. 3) in all participants. We found negative associations between listening effort and grey matter volume in the cerebellum and brain stem as well as the in orbitofrontal cortex (BA 11) and the inferior frontal cortex (BA 47). Cortical thickness in the right orbitofrontal cortex (BA 12) also correlated negatively with listening effort (MNI coordinate *x* = 5, *y* = 58, *z* = − 16; cluster size *k* = 137; *z*-value = 3.51).

**Table 1:** Peak coordinates for the grey matter volume regression analysis with listening effort

Peak coordinates (*x*, * y*, * z*)	*Z*-value	Cluster size	Brain region
− 23, − 71, − 48	4.62	2038	Cerebellum, left
5, − 12, − 48	4.26	297	Ventral DC, right
8, 41, − 33	4.27	529	Orbitofrontal cortex, right
− 45, 41, 6	4.25	394	Inferior frontal gyrus, left
21, − 68, − 26	3.69	394	Cerebellum, right
15, − 68, − 50	3.64	820	Cerebellum, right
− 26, − 56, 29	3.61	357	Cerebellum, left

### DTI data

To investigate white matter integrity, mean FA values from tracts connecting auditory, frontal and visual areas—such as in the superior and inferior fronto-occipital and longitudinal fasciculi as well as in the cingulum and uncinate fasciculus—were computed. Mean values for hard-of-hearing and normal-hearing participants can be seen in Table 2. Neither significant differences between both groups nor significant correlations between FA values and self-rated daily life listening effort across all participants were obtained after correction for multiple comparisons. To provide evidence for the absence of changes in FA values, we computed an additional Bayesian independent samples *T* test with default prior option (Cauchy distribution) comparing FA values between both groups. This analysis showed evidence for the null hypothesis in most of the brain regions, except the cingulum (left middle cingulum: BF_10_ = 5.993; moderate evidence for the alternative hypothesis). Statistical outcome values can be seen in Table 2 (*p* values, effect sizes and Bayes factors). The Bayesian correlation matrix with FA values and self-rated listening effort showed evidence for the null hypothesis in all brain regions (all BF_10_ < 1). Note that a post hoc analysis of another common DTI measure, mean diffusivity (data not shown), did not provide any evidence for microstructural changes related to hearing loss or listening effort in the investigated tracts.

**Table 2:** Fractional anisotropy values for hard-of-hearing and normal-hearing individuals

Region	Hard-of-hearing	Normal hearing	Significance [*p* value]	Effect size [Cohen’s * d*]	BF_10_
Cingulum anterior L	0.171 (± 0.017)	0.176 (± 0.015)	0.227	− 0.295	0.743
Cingulum anterior R	0.181 (± 0.019)	0.181 (± 0.016)	0.906	0.029	0.254
Cingulum mid L	0.169 (± 0.018)	0.179 (± 0.014)	0.011	− 0.606	5.993
Cingulum mid R	0.192 (± 0.019)	0.199 (± 0.019)	0.110	− 0.39	1.109
Cingulum posterior L	0.252 (± 0.061)	0.277 (± 0.063)	0.103	− 0.399	1.081
Cingulum posterior R	0.324 (± 0.075)	0.353 (± 0.065)	0.092	− 0.414	1.191
Uncinate fasciculus R	0.234 (± 0.015)	0.230 (± 0.012)	0.249	0.282	0.398
Uncinate fasciculus L	0.234 (± 0.016)	0.228 (± 0.016)	0.122	0.377	0.825
Inferior longitudinal fasciculus R	0.220 (± 0.013)	0.220 (± 0.012)	0.997	0.001	0.259
Inferior longitudinal fasciculus L	0.224 (± 0.012)	0.224 (± 0.012)	0.911	− 0.027	0.254
Superior longitudinal fasciculus R	0.211 (± 0.011)	0.210 (± 0.011)	0.825	0.054	0.27
Superior longitudinal fasciculus L	0.214 (± 0.012)	0.215 (± 0.013)	0.817	− 0.056	0.266
Inferior fronto-occipital fasciculus R	0.260 (± 0.013)	0.260 (± 0.014)	0.967	− 0.01	0.255
Inferior fronto-occipital fasciculus L	0.273 (± 0.013)	0.270 (± 0.013)	0.209	0.306	0.453
Superior fronto-occipital fasciculus L	0.477 (± 0.044)	0.467 (± 0.039)	0.345	0.23	0.314
Superior fronto-occipital fasciculus L	0.439 (± 0.039)	0.430 (± 0.043)	0.378	0.214	0.327

Mean values and standard deviation; significance for two-sample *t* test between groups; Bonferroni corrected significance threshold *p* = 0.003; BF_10_ indicates the Bayes factor in favour of H_1_ over H_0_

## Discussion

In a large sample of elderly subjects with age-appropriate hearing and subjects with untreated mild to moderate age-related high-frequency hearing loss, we investigated changes in grey and white matter. Furthermore, the influence of self-rated daily life listening effort on these structural brain alterations was examined. Our results showed decreased grey matter volume in the middle frontal gyrus in hard-of-hearing compared to normal-hearing participants. Additionally, we obtained a negative relation between listening effort and grey matter volume as well as cortical thickness in several prefrontal brain regions. However, our DTI data analysis revealed no significant changes in white matter integrity in age-related hearing loss or any associations with daily life listening effort. The additional Bayesian approach only showed moderate support for differing FA values between both groups in the left middle cingulum.

### Grey matter volume changes in age-related hearing loss

We expected a decrease in grey matter volume and cortical thickness in temporal and prefrontal brain regions in hard-of-hearing compared to normal-hearing participants. Our results showed significantly lower grey matter volume in middle frontal gyrus in hard-of-hearing compared to normal-hearing participants, but no significant differences in cortical thickness.

Previous research has shown grey matter decreases in cingulate, superior temporal, superior and medial frontal gyri along with the occipital lobe and hypothalamus in hard-of-hearing compared to normal-hearing participants (Boyen et al. 2013; Husain et al. 2011). However, the focus in these studies was to disentangle neuroanatomical changes due to tinnitus and hearing loss and not to investigate the structural changes between hard-of-hearing and normal-hearing participants (as is the focus of the current study). Others have shown correlations between grey matter volume and cortical thickness in frontal brain regions—including the middle frontal gyrus—with speech perception in elderly participants (Giroud et al. 2018; Rudner et al. 2019; Wong et al. 2010). Task-based neuroimaging studies showed that increased activity in the middle frontal gyrus seems to be associated with effortful listening in hard-of-hearing individuals (Rosemann and Thiel 2018) as well as in normal-hearing elderly participants (Erb and Obleser 2013). In addition, the left middle frontal gyrus plays a role in phonemic perception (Raizada and Poldrack 2007). Hence, there is evidence that the middle frontal gyrus is associated with speech perception—particularly in difficult listening conditions—and that grey matter loss in the frontal lobe is associated with worse speech perception. We here present significantly decreased grey matter volume in the middle frontal gyrus in mild to moderate age-related hearing loss. This is the first study assessing neuroanatomical correlates in a large sample of presbycusis and suggests that a decrease in hearing abilities is related to a loss of grey matter volume in a brain region associated with speech perception. How this relates to speech perception in general or more specifically to speech in noise perception needs to be further explored in future research.

In contrast to our expectations, we did not find any grey matter loss in auditory regions as indicated by previous research (Armstrong et al. 2018; Eckert et al. 2012, 2019; Qian et al. 2017). Possible reasons for that may be that in these studies, also participants up to the age of 88 years were included, whereas our sample included individuals up to the age of 74 years. Hence, the hearing loss in our sample might have lasted for a shorter duration and therefore might not have led to a decrease in grey matter volume within the auditory cortex at this stage. Further, the younger age in our sample may have allowed for a better compensation of the only mild to moderate hearing impairment than that would be the case at older age.

### Alterations in grey matter volume and cortical thickness related to listening effort

Increased listening effort in elderly participants was recently associated with a decrease in resting state functional connectivity between the auditory and inferior frontal cortex (Rosemann and Thiel 2019). Therefore, we also investigated the impact of self-rated listening effort on changes in grey matter volume and cortical thickness. We hypothesized that an increased daily life listening effort is associated with a decrease in grey matter volume and cortical thickness in the prefrontal cortex. Our analyses confirmed the negative correlations between listening effort and grey matter volume and cortical thickness in the right orbitofrontal cortex along with grey matter volume in the left inferior frontal cortex.

The structural alterations we report here are consistent with previous task-based fMRI studies showing increased neural activity in the frontal cortex covering inferior frontal, middle frontal and cingular–opercular brain regions in demanding listening conditions (Davis and Johnsrude 2003; Erb and Obleser 2013; Rosemann and Thiel 2018; Wild et al. 2012; Wong et al. 2009). This increased frontal lobe recruitment is supposed to reflect effortful listening not only in hard-of-hearing, but also normal-hearing listeners. In contrast, we here assessed self-rated listening effort in everyday life situations as well as associated changes in grey matter volume and cortical thickness. Using the same measure of self-rated listening effort, we previously found a link between self-rated listening effort and a decrease in resting state functional connectivity between auditory and left inferior frontal cortex (Rosemann and Thiel 2019). Hence, a link between increased listening effort and inferior frontal cortex activity as well as connectivity has previously been established by functional MRI studies. We now add further evidence that increased self-rated daily life listening effort is also related to grey matter volume loss in the left inferior frontal cortex. In addition, we found changes in the right orbitofrontal cortex, which plays a role in emotional evaluation and judgements (Schirmer and Kotz 2006; Wildgruber et al. 2009) along with guessing (Elliot et al. 1999). Moreover, there is evidence that the orbitofrontal cortex is involved in target detection in dichotic listening (Pollmann et al. 2004) and in processing emotional voice stimuli (Blasi et al. 2011). Thus, the orbitofrontal cortex is related to decision-making processes, such as selection and evaluation of stimuli including auditory and speech input. However, the orbitofrontal cortex also plays a key role in emotional processes, especially by representing the expected reward values (Rolls 2019). The assessment of self-rated listening effort involves the conscious evaluation of the mental effort that was required or perceived during daily life listening situations (Francis and Love 2020). This evaluation is also corroborated by the listener’s feelings and expectations about the expended listening effort (Francis and Love 2020). The current study adds evidence of cortical thinning and grey matter volume loss in the orbitofrontal cortex in subjects reporting higher listening effort. Hence, structural changes in orbitofrontal cortex are probably related to the conscious evaluation along with the expectation of the increased self-rated listening effort rather than to the attentional and cognitive resource allocation process (Francis and Love 2020).

In contrast to cortical volume, which differed in the prefrontal regions between hard-of-hearing and normal-hearing participants, changes in cortical thickness were only observed in relation to listening effort. A recent study demonstrated that cortical thinning was closely related to speech perception abilities (Giroud et al. 2018). Other studies suggest not only evaluating hearing abilities by simple pure-tone audiograms, but also outcome measures such as speech-in-noise perception as these may be better indicators of the experienced difficulties due to the decreased auditory input (Cardin 2016; Humes et al. 2013b; Lin 2012; Moore et al. 2014). The assessment of self-rated listening effort offers an easy evaluation of the experienced mental effort during demanding listening situations (Bernarding et al. 2017; Pichora-Fuller et al. 2016) and hence includes listening to speech rather than pure tones and a cognitive component. Additionally, the use of self-rated listening effort questionnaires may enable to directly assess the listeners’ conscious awareness of their listening effort and therefore may also be relevant in clinical applications (Johnson et al. 2015). When speech and sounds are audible, listening can still be effortful, even stressful and tiring, with the consequence that individuals quit listening (Pichora-Fuller et al. 2016). Preventing individuals from avoiding effortful listening situations is therefore highly important.

In addition, the assessment of cortical thickness has recently gained importance in dementia screening (Hartikainen et al. 2012; Schwartz et al. 2016). This is mainly because cortical thickness is not related to total intracranial volume, which may vary across age, sex and brain regions (Im et al. 2008; Neth et al. 2020). Hence, previous work suggests using cortical thickness measures as a more reliable assessment of neurodegeneration (Giroud et al. 2018; Hartikainen et al. 2012; Neth et al. 2020; Schwartz et al. 2016). In combination, our study showed that there is a close relationship between self-rated listening effort and cortical thickness in the orbitofrontal cortex. Both measures may become relevant in determining neuroanatomical mechanisms in age-related hearing loss potentially benefitting hearing-aid treatment. Cortical thickness has already gained attention as a biomarker in Alzheimer’s disease (Bakkour et al. 2009; Dickerson and Wolk 2012). In elderly participants, cortical thinning may be used as an indicator for neural consequences of increased listening effort that may require hearing-aid treatment. Elderly individuals experiencing a relative decrease in hearing abilities (not classified as hearing impairment) may already report high listening effort and therefore avoid demanding listening situations. Probably, these individuals already benefit from hearing-aid fitting to decrease the enhanced listening effort. However, intervention studies are needed to investigate this issue in more detail. These intervention studies may offer valuable insights into how neuroanatomical changes in age-related hearing loss are related to hearing-aid outcome.

In summary, our results strengthen the assumption that grey matter loss and cortical thinning in inferior frontal and orbitofrontal brain regions are associated with an increased self-rated daily life listening effort. Since research regarding changes in cortical thickness in age-related hearing loss is entirely lacking up to now, these findings may become particularly relevant in determining neuroanatomical consequences of hearing loss and possibly in predicting hearing-aid outcome.

### No evidence for influence of hearing abilities or listening effort on white matter integrity

Contrary to our expectations, we found no evidence for changes in FA values in the superior and inferior fronto-occipital and longitudinal fasciculi as well as in the cingulum and the uncinate fasciculus as a function of hearing loss or listening effort. The additional Bayesian *T* test showed evidence for the null hypothesis in most of the brain regions and moderate support for differing FA values between hard-of-hearing and normal-hearing individuals in the left middle cingulum. A few previous studies found significantly lower FA values in hard-of-hearing compared to normal-hearing participants (Husain et al. 2011; Ma et al. 2016; Luan et al. 2019b). However, in the first study, the sample size was rather small (*n* = 7 hard-of-hearing; *n* = 11 normal hearing) and statistical thresholds were more liberal than in our study (*p* < 0.1; corrected for multiple comparisons; Husain et al. 2011). The second study investigated presbycusis participants with similar hearing curves and age as in our study; however, the sample size was also small (*n* = 15; Ma et al. 2016). In the last study, the focus was on long-term bilateral sensorineural hearing loss and included individuals with tinnitus (Luan et al. 2019b). These differences in study population and size may explain the discrepant findings. We are the first study investigating FA values in a large homogenous sample of elderly participants (*n* = 71) with normal hearing as well as mild to moderate age-related hearing loss. The findings in this sample do not indicate a relation between FA values and self-reported daily life listening effort. The Bayesian approach showed a moderate support for differing FA values between hard-of-hearing and normal-hearing participants in the left cingulum, but not in any other brain region. A possible explanation might be that the mild to moderate decrease in hearing abilities and the slight increase in listening effort were not advanced enough to lead to structural changes in white matter metrics such as FA values. Hearing loss in old age is a gradual, slowly progressing process which often remains undiscovered for several years. Accordingly, analyses investigating the impact of the duration of hearing loss on white matter changes are challenging, but would be important to determine whether more severe stages or an extended period of age-related hearing loss is associated with changes in white matter structures. Future longitudinal research may address this question.

## Conclusion

We investigated the neuroanatomical correlates of age-related hearing loss in a large group of elderly normal-hearing and hard-of-hearing participants. Our results showed significantly lower grey matter volume in the middle frontal cortex in hard-of-hearing participants. Furthermore, higher self-rated listening effort was associated with lower grey matter volume in the inferior and orbitofrontal cortex along with lower cortical thickness in the orbitofrontal cortex. In contrast, white matter integrity was not related to age-related hearing loss or daily life listening effort. Hence, we provide evidence that age-related hearing impairment as well as daily life listening effort seems to be associated with grey matter loss in the prefrontal brain regions. Alterations in cortical thickness seem to be linked to the increased listening effort rather than the hearing loss itself. This implies that not only hearing abilities, but also behavioural outcome measures of the decreased auditory input—such as self-rated daily life listening effort—need to be assessed and evaluated with regard to structural alterations in elderly participants. These findings advance our knowledge of underlying structural brain changes in age-related hearing loss. An important question to be addressed in future studies is whether these can be halted or reversed, for instance, by hearing-aid fitting.

## Data Availability

The data that support the findings of this study are available upon reasonable request from the corresponding author. The data are not publicly available owing to potentially identifying information that could compromise participant privacy.
